# The impact of recognition, fairness, and leadership on employee outcomes: A large-scale multi-group analysis

**DOI:** 10.1371/journal.pone.0312951

**Published:** 2025-01-09

**Authors:** Hyeon Jo, Donghyuk Shin

**Affiliations:** 1 Headquarters, HJ Institute of Technology and Management, Bucheon, Gyeonggi-do, Republic of Korea; 2 Headquarters, Secufind Co., Seoul, Republic of Korea; University of Valencia: Universitat de Valencia, SPAIN

## Abstract

In the dynamic field of organizational behavior, comprehending the determinants of employee engagement, burnout, and job satisfaction is pivotal. This research investigates the influence of various workplace factors, such as recognition, fairness, leadership, and workload, on these key employee outcomes. Utilizing Partial Least Squares Structural Equation Modeling (PLS-SEM) for analysis, the study examines data from 25,285 employees. The results indicate that recognition significantly boosts employee engagement, while fairness and involvement also positively contribute, albeit to a lesser extent. Transformational leadership plays a dual role, enhancing engagement and reducing burnout. Notably, workload overload presents a nuanced impact, affecting both engagement and burnout. The study additionally reveals the detrimental effect of technological disruption anxiety on job satisfaction. A significant finding from the Multi-Group Analysis (MGA) is the varying impact of these factors between the private and public sectors, particularly in the context of transformational leadership’s effect on burnout and the differential influence of workload on burnout. These insights are critical for formulating effective organizational strategies and policies, highlighting the need for customized recognition initiatives, equitable management approaches, and well-balanced workload allocation.

## Introduction

Regular workers, often the mainstay of the modern workforce, play a crucial role in driving economic growth and societal stability [1]. As workers navigate the intricacies of balancing work and personal life in a rapidly evolving and often high-pressure environment, their job satisfaction and overall well-being become critical not only to their personal fulfillment but also to broader organizational and societal outcomes. The level of satisfaction these workers experience in their roles significantly influences their productivity, engagement, and loyalty to their employers, thereby directly affecting organizational performance and sustainability [2, 3]. Moreover, their well-being has far-reaching implications, extending beyond the workplace to impact social structures, community health, and economic vitality [4]. As such, understanding and enhancing the job satisfaction of regular workers is essential not just for organizational success, but for fostering a thriving, resilient workforce that can adeptly meet the challenges of a dynamic global economy.

Organizational culture is a pivotal factor in shaping the attitudes and outcomes of regular workers, significantly influencing their job satisfaction and overall engagement. Key elements like recognition, fairness, involvement, and transformational leadership are essential in creating a positive work environment. Recognition addresses employees’ intrinsic need for esteem and belonging, playing a vital role in enhancing job satisfaction and boosting morale [5]. Fairness, or perceived organizational justice, not only impacts job satisfaction but also instills a sense of respect and value among employees, thereby fostering a more harmonious and productive workplace [6]. Employee involvement in decision-making processes contributes significantly to job satisfaction by promoting a sense of ownership and empowerment, leading to increased commitment and productivity [7]. Furthermore, transformational leadership, characterized by its ability to inspire and motivate employees, is directly linked to higher levels of job satisfaction. Such leaders focus on employee development and well-being, creating a supportive and stimulating work environment that encourages personal and professional growth [8, 9]. These cultural elements collectively create a work atmosphere conducive to employee satisfaction, which is critical for retaining talent and ensuring the long-term success of an organization.

Overload and competition stand out as significant determinants of job satisfaction. Overload, reflecting the degree to which work demands surpass an employee’s capacity, commonly results in job dissatisfaction, primarily due to escalated stress and the onset of burnout [10]. In contrast, workplace competition exhibits a dual nature in influencing employee satisfaction. When competition is moderate, it can positively impact job satisfaction by promoting a sense of accomplishment and driving motivation. However, when competition reaches excessive levels, it tends to have the opposite effect, often leading to increased stress and resulting in overall job dissatisfaction [11]. This highlights the delicate balance that organizations must maintain in managing workloads and fostering a competitive yet supportive work environment.

Understanding job satisfaction requires examining key psychological factors, including work engagement, burnout, and technological disruption anxiety. Work engagement, marked by high levels of vigor, dedication, and absorption, is closely linked to enhanced job satisfaction. Engaged employees, who are energetically involved in their tasks, generally report higher levels of satisfaction in their work [3]. Conversely, burnout, particularly its exhaustion aspect, is a significant detractor from job satisfaction. Employees experiencing burnout often feel drained and overwhelmed, leading to diminished satisfaction and productivity [12]. Additionally, the growing anxiety associated with technological disruption and the uncertainties it brings, such as job insecurity, has a notable negative impact on job satisfaction. As technology rapidly changes job landscapes, the ensuing anxiety can contribute to a decrease in employees’ overall contentment with their work [13]. This complex interplay of engagement, burnout, and technological anxiety highlights the multifaceted nature of job satisfaction in the modern workplace.

Despite extensive research in these areas, gaps remain in understanding the combined effect of these variables on job satisfaction, particularly in a large-scale context across different sectors. This paper aims to address these gaps by conducting a comprehensive analysis involving a substantial sample size, thus providing more generalizable results. Specifically, it investigates the differential impacts of organizational culture, workload, and emerging challenges on job satisfaction among regular workers in the private and public sectors.

This study distinctively contributes to organizational behavior literature by examining a comprehensive set of factors within a large-scale, multi-sector framework, thus providing a broader generalization of results across different work environments. A significant novelty of this research is the incorporation of the relatively new job demand of technological disruption anxiety, reflecting the current era’s rapid technological changes and their psychological impacts on employees [13]. Additionally, the use of Multi-Group Analysis (MGA) allows for nuanced comparisons between private and public sectors, highlighting sector-specific dynamics that previous studies often overlook. These elements enrich the theoretical foundations laid by the Job Demands-Resources model (JD-R) by introducing and testing counterintuitive hypotheses concerning the positive effects of certain job demands on job satisfaction [14]. This large-scale study not only adds to the extant literature by updating it with contemporary job demands but also challenges and expands existing theories to include new and emerging workplace challenges, providing both scholarly and practical insights into managing workforce dynamics in the face of ongoing digital transformation.

The structure of this paper encompasses a review of relevant literature in the ensuing section, followed by the theoretical foundation and hypotheses in the Theoretdcial background and research hypotheses section. Research methodology section delves into the research methodology employed, while the Analysis and results section 4 presents the empirical findings. A thorough discussion of these findings is laid out in the Discussion section. Finally, the Conclusion section concludes the paper with an exploration of theoretical contributions, practical implications, study limitations, and potential avenues for future research.

## Theoretical background and research hypotheses

**Fig 1:**
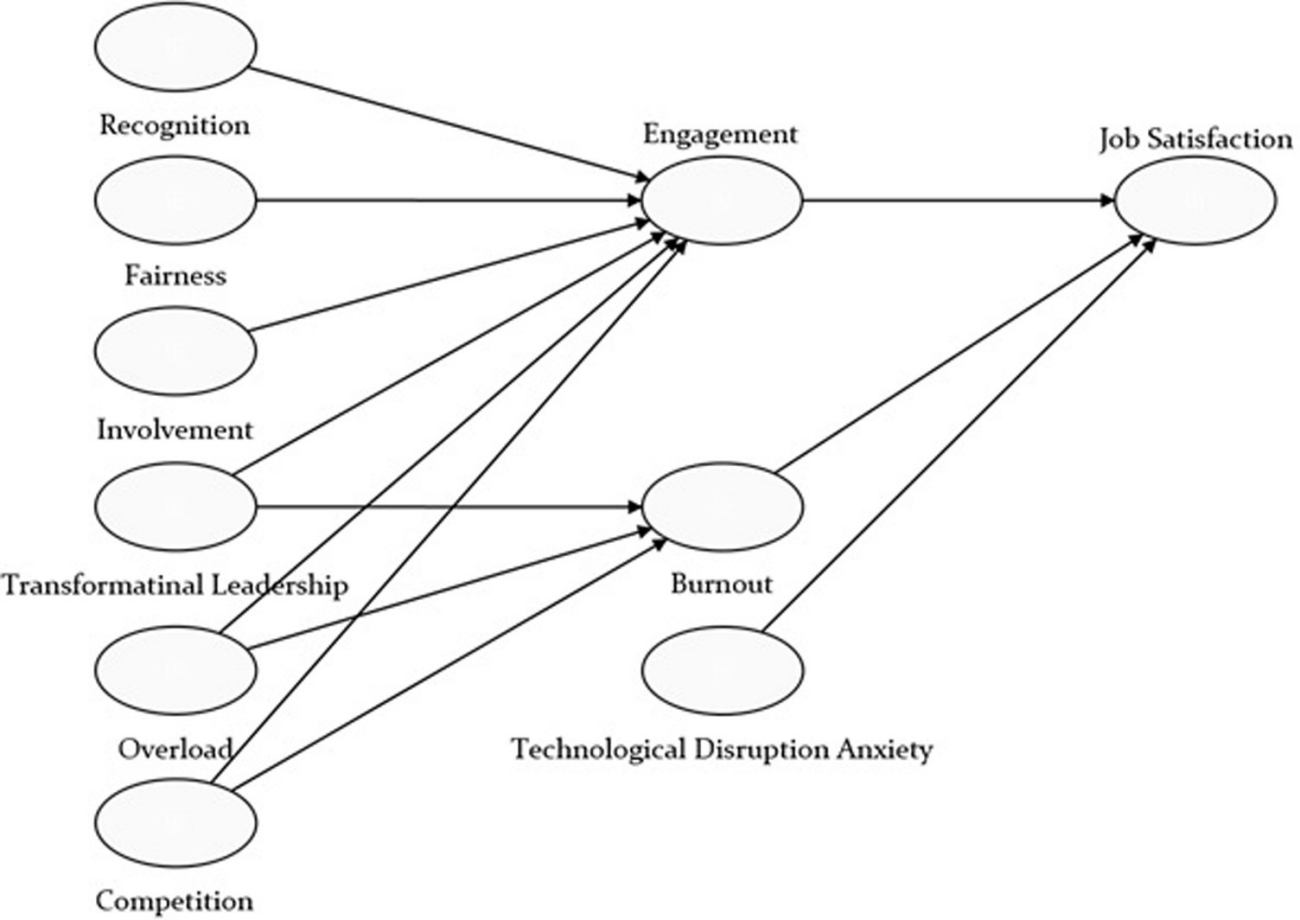
Research model.

This study proposes a comprehensive model grounded in the JD-R theory [14]. This theory provides a robust framework for understanding how various workplace elements interact to influence employee outcomes, such as job satisfaction, engagement, and burnout. The JD-R theory posits that job demands (e.g., workload, competition) and job resources (e.g., recognition, fairness, involvement, transformational leadership) have distinct and interrelated effects on employee well-being and organizational outcomes. According to this theory, job demands are physical, psychological, social, or organizational aspects of the job that require sustained effort and are associated with certain physiological and psychological costs (e.g., burnout). In contrast, job resources refer to those physical, psychological, social, or organizational aspects of the job that are functional in achieving work goals, reduce job demands and the associated costs, and stimulate personal growth, learning, and development (e.g., engagement). Our research model integrates these concepts by examining how specific job demands (overload and competition) and resources (recognition, fairness, involvement, and transformational leadership) contribute to outcomes such as engagement, burnout, and ultimately, job satisfaction. Additionally, we incorporate the concept of technological disruption anxiety as a modern job demand, reflecting the changing nature of work and its impact on employee well-being. Fig 1 shows the research model of this study.

### Recognition

Recognition in the workplace is identified as a potent intrinsic motivator that not only alleviates job dissatisfaction but actively fosters job satisfaction and propels productivity [15–17]. Herzberg [18]’s Two-Factor Theory posits that job satisfaction and dissatisfaction are driven by different sets of factors—motivators and hygiene factors, respectively, with recognition falling into the category of motivators that directly enhance employee morale and output. Further empirical support is provided by previous studies, which highlight recognition as a crucial workplace resource that elevates employee engagement [19–21]. Moreover, several studies argue that recognition feeds into an employee’s sense of achievement and perceived value within the organization, effectively enhancing their engagement levels [22, 23]. This is particularly relevant in the context of the JD-R model, which delineates how resources like recognition counterbalance job demands and augment employee engagement by improving emotional and psychological well-being [14, 24]. Thus, integrating these theoretical and empirical insights, this study formulates the following hypothesis:

H1. Recognition has a positive effect on engagement among regular employees.

### Fairness

F Fairness in the workplace, encompassing employees’ perceptions of equity and justice, is pivotal in shaping organizational dynamics [6, 25]. It aligns with Adams’s Equity Theory, which posits that fairness perceptions directly influence employee motivation and satisfaction, laying the groundwork for higher organizational commitment and performance [26]. Extensive research has validated this relationship, with studies demonstrating that perceived fairness correlates with a range of positive employee outcomes, such as enhanced job satisfaction, increased trust, and greater engagement [27–32]. Empirical evidence further underscores the impact of fairness on engagement, illustrating that fairness not only promotes general work satisfaction but also fosters organizational citizenship behaviors, a key indicator of deep engagement [33, 34]. Such behaviors often translate into employees going beyond their formal job requirements, contributing to organizational success. As well, research reveal that fairness in reward distribution and procedural transparency significantly boosts these behaviors by reinforcing a sense of value and respect among employees [35, 36]. Therefore, integrating these insights, this study hypothesizes that perceived fairness within the workplace significantly enhances employee engagement.

H2. Fairness has a positive effect on engagement.

### Involvement

Involvement in the workplace, characterized by employee participation in decision-making processes, is a critical factor in enhancing organizational commitment and job satisfaction [37]. Research has consistently demonstrated that when employees are involved in aspects that affect their work life, their job satisfaction and commitment to the organization increase significantly [2, 38–41]. This active participation not only improves their satisfaction but also serves as a catalyst for intrinsic motivation, empowering employees and fostering deeper engagement with their roles [42–44]. Further extending this concept, involvement is also identified as a critical mechanism for enhancing psychological empowerment [45, 46]. When employees feel empowered, they exhibit higher levels of initiative and engagement, which are vital for organizational success [47, 48]. Empowered employees are more likely to take ownership of their tasks and responsibilities, contributing positively to the organizational climate and achieving higher performance outcomes. Given these insights, the hypothesis proposed in this study is that greater employee involvement in organizational processes leads to higher levels of engagement by fostering psychological empowerment and intrinsic motivation.

H3. Involvement has a positive effect on engagement.

### Transformational leadership

Transformational leadership is a dynamic leadership style where leaders inspire and motivate employees beyond their self-interests for the organization’s benefit [49]. This approach, distinguished by its four dimensions—idealized influence, inspirational motivation, intellectual stimulation, and individualized consideration—has been robustly associated with higher employee engagement [50]. Numerous studies have demonstrated how transformational leaders enhance engagement by fostering an empowering and motivating work environment that encourages employees to excel and commit to organizational goals [8, 51–56]. Furthermore, the role of transformational leadership in mitigating employee burnout addresses how it can significantly reduce workplace stressors [57, 58]. Burnout results from chronic stress in the workplace [59, 60], which transformational leaders can alleviate by creating a supportive and stimulating work environment [8, 61]. These leaders reduce burnout by decreasing role ambiguity and workload, essential stressors identified in the literature [14, 62–64]. By integrating these elements, the following hypotheses are proposed in this study, exploring the dual impact of transformational leadership on enhancing engagement and reducing burnout among employees.

H4a. Transformational leadership has a positive effect on engagement.
H4b. Transformational leadership has a negative effect on burnout.

### Overload

Overload, defined as an excessive workload or exceedingly high demands in the workplace, is generally recognized as a significant stressor [10, 65]. The extensive literature on job demands posits that such overload can deplete employee energy, leading directly to reduced levels of engagement [66, 67]. Conversely, overload has been associated with increased burnout, particularly due to its capacity to exhaust an individual’s mental and physical resources [68–70]. Specifically, it contributes to the exhaustion component of burnout by persistently straining the employee beyond their resilience and recovery capabilities [14, 71, 72]. This body of work establishes a clear theoretical foundation for exploring how high job demands not only hinder engagement by taxing employees’ capacities but also facilitate burnout by overwhelming their ability to cope with stress. Thus, this study suggests the following hypotheses.

H5a. Overload has a negative effect on engagement.
H5b. Overload has a positive effect on burnout.

### Competition

Competition in the workplace, often seen as a motivator, can have varying effects on employee outcomes [73–75]. According to goal-setting theory [76], competition can enhance engagement by setting clearer targets and fostering a sense of achievement. A competitive climate can stimulate motivation and productivity, potentially leading to higher engagement [23, 74, 77]. However, the relationship between competition and burnout is more straightforward. As detailed in the JD-R model [14], competition, as a job demand, can increase stress, potentially leading to burnout [3, 74, 78]. Research supports this, showing how competitive pressures can contribute to emotional exhaustion, a key dimension of burnout [70, 79, 80]. Therefore, while competition might foster engagement through goal achievement and motivation, it also has the potential to increase burnout due to heightened stress and pressure. Thus, this study suggests the following hypotheses.

H6a. Competition has a positive effect on engagement.
H6b. Competition has a positive effect on burnout.

### Engagement

Engagement reflects a positive, fulfilling work-related state of mind characterized by vigor, dedication, and absorption [3, 81]. This state of being highly involved and enthusiastic about one’s work has been linked to increased job satisfaction [82–85]. Studies demonstrate a positive correlation between high levels of engagement and greater job satisfaction, suggesting that when employees are deeply engaged in their work, they tend to experience higher levels of overall job satisfaction [14, 86–89]. Thus, this study suggests the following hypothesis.

H7. Engagement has a positive effect on satisfaction.

### Burnout

Burnout comprises emotional exhaustion, depersonalization, and a reduced sense of personal accomplishment [12, 90]. This state of chronic workplace stress is negatively correlated with job satisfaction [91–93]. Studies demonstrates that as burnout increases, especially in terms of emotional exhaustion and depersonalization, job satisfaction tends to decrease significantly [70, 94]. This inverse relationship is also supported by the JD-R model [14], which posits that excessive job demands leading to burnout can deplete an employee’s psychological resources, resulting in lower job satisfaction. Furthermore, Schaufeli and Bakker [3] have highlighted that burnout negatively impacts an individual’s attitude towards their job, thereby reducing their overall job satisfaction. Thus, this study suggests the following hypothesis.

H8. Burnout has a negative effect on satisfaction.

### Technological disruption anxiety

Technological disruption anxiety encompasses concerns about job security, changes in job roles, and adaptability due to technological advancements [95]. It can lead to increased stress and uncertainty among employees. The Conservation of Resources theory [96] supports this, suggesting that the potential loss of resources (like job security or familiar work routines) can result in decreased job satisfaction. Moreover, Sverke and Hellgren [97] shows that job insecurity, a key component of technological disruption anxiety, is strongly correlated with reduced job satisfaction. This is further corroborated by studies examining the psychological impacts of technological changes in the workplace [13], which find that anxiety related to these changes can lead to lower levels of overall satisfaction with one’s job. Thus, this study suggests the following hypothesis.

H9. Technological disruption anxiety has a negative effect on satisfaction.

## Research methodology

Since this study involved the analysis of secondary data, it was exempt from review. Exemption from review was obtained from the Public Institutional Review Board Designated by Ministry of Health and Welfare (IRB) (Approval No: P01-202404-01-035). In compliance with the retrospective study requirements, the data for this research were accessed on April 17, 2024. The data provider had anonymized the data prior to our access, ensuring that no information available to the authors could identify individual participants during or after data collection. This anonymization process ensures the confidentiality and privacy of all participants involved in the study. The data analyzed in this study can be downloaded from the following sites.

https://oshri.kosha.or.kr/oshri/researchField/downWorkingEnvironmentSurvey.do

### Research design and paradigm

The research design of this study is grounded in a pragmatic paradigm, which focuses on the practical application of findings and the value of outcomes to address specific problems [98]. This approach supports the use of mixed methodologies, aligning well with our employment of Partial Least Squares Structural Equation Modeling (PLS-SEM) to analyze complex models and relationships within organizational behavior [99]. The selection of PLS-SEM was driven by its robustness in handling complex model structures and its efficacy with smaller sample sizes, making it suitable for the intricacies of behavioral research [99]. Our sampling strategy, informed by Rahi [98]’s review on sampling issues, ensured a comprehensive representation across various sectors, enhancing the generalizability of the findings. The instrument development was meticulously undertaken, drawing from established scales and items tailored to the constructs of interest, ensuring reliability and validity in capturing the dynamics of employee engagement, satisfaction, and burnout.

### Instrument

This study was conducted based on data from the 6th Korean Working Conditions Survey (KWCS), carried out during 2020–2021. The scales were developed using items collected from this survey and were informed by related research.

For measuring recognition, a branching question was posed under the premise, "How is your workplace in the following aspects?" An example item is "Employees receive recognition and praise when they do their job well," measured on a scale from ‘strongly agree’ (1) to ‘strongly disagree’ (5).

An example item for fairness is "Conflicts are resolved in a fair manner," also measured on the same 5-point scale. Involvement was assessed with items such as "I am involved in improving the structure of my department, organization, or work procedures," using a scale from ‘always’ (1) to ‘never’ (5). Transformational leadership was measured based on respondents’ perceptions of their immediate supervisors, with an example item being "My immediate supervisor respects me personally," using the same 5-point scale. Overload was assessed with two items under the question "How often do you experience the following situations during your working hours?" Examples include "Working at a very fast pace" and "Working to meet strict deadlines," measured on a scale from ‘always’ (1) to ‘never’ (7). Competition was measured with the item "I feel that I am in fierce competition with others in my work," using the 5-point scale. Engagement and burnout were measured starting with the root question "How often do you experience the following emotions while working?" measured from ‘always’ (1) to ‘never’ (5). An example item for engagement is "I feel full of energy when I work," and for burnout, "I feel physically exhausted when I finish work." Technological disruption anxiety was assessed with items like "I am worried about a situation where my income decreases due to technological progress and automation," measured from ‘very worried’ (1) to ‘not worried at all’ (4). Satisfaction was measured with the question "Are you satisfied with your overall working environment?" on a scale from ‘very satisfied’ (1) to ‘not satisfied at all’ (4). For interpretive consistency, the indicators in this study were reverse coded. The specific contents and related studies are detailed in Table 1.

**Table 1:** List of constructs and items.

Construct	Items	Measure	Related Work
Recognition	RCN1	Considering the effort and achievements in my work, I am receiving appropriate compensation.	Herzberg [100] ;Bassett‐Jones and Lloyd [101]
	RCN2	I receive appropriate recognition for my work.	
	RCN3	Employees receive recognition and praise when they do their job well.	
Fairness	FAR1	Conflicts are resolved in a fair manner.	Greenberg [6] ;Colquitt, Conlon [102]
	FAR2	Work is distributed fairly.	
	FAR3	I am treated fairly at work.	
Involvement	IVV1	I am asked for my opinion before work goals are determined.	Lawler III [7] ;Spreitzer [103]
	IVV2	I am involved in improving the structure of my department, organization or work procedures.	
	IVV3	I can reflect my thoughts when I do my work.	
Transformational Leadership		How is your immediate supervisor in the following aspects? Please select the appropriate response for each item.	Bass and Riggio [104] ;Top, Akdere [105] ;Wan Omar and Hussin [106]
	TFL1	I am respected personally.	
	TFL2	Useful advice (feedback) is given about the work.	
	TFL3	I am encouraged and helped to develop.	
Overload		How often do you experience the following situations during your working hours?	Karasek Jr [10] ;Sonnentag and Frese [107]
	OVL1	Working at a very fast pace.	
	OVL2	Working to meet strict deadlines.	
Competition	COM1	I feel like I’m competing fiercely with others in terms of work.	Pfeffer and Leblebici [108] ;Lee and Yang [109]
Engagement	EGM1	I feel full of energy when I work.	Schaufeli, Bakker [110] ;Schaufeli and Bakker [3]
	EGM2	I am passionate in my work.	
	EGM3	Time flies when I work.	
Burnout	BUR1	I feel physically exhausted when I finish work.	Han and Kwak [111] ;Schaufeli and Bakker [3]
	BUR2	I feel mentally exhausted from work.	
Technological Disruption Anxiety		How concerned are you about the potential impact of technological advancements and automation on your work in the future, considering these situations:	Frey and Osborne [95]
	ANX1	A situation in which income is decreasing.	
	ANX2	A situation in which an organization does not have interest in doing things contrary to my expectations.	
	ANX3	A situation in which working hours are different from my expectations.	
Job Satisfaction	SAT1	Are you satisfied with your overall working environment?	Ashraf [112] ;Wan Omar and Hussin [106]

In this study, the decision to use only one item for measuring both competition and job satisfaction was significantly influenced by the constraints of the KWCS, which employs single-item measures for these variables. This approach, while seemingly simplistic, is grounded in the principles of methodological rigor and efficiency associated with PLS-SEM. Adhering to the guidelines that suggest single-item measures can be valid and reliable when the construct is concrete and narrowly scoped [113], this method is employed. Additionally, this choice is consistent with survey design considerations where brevity is known to enhance response rates and minimize respondent fatigue, thereby maintaining the integrity and validity of the research findings [114]. This study considers gender and age as control variables.

### Data

This study utilized data from the 6th KWCS, conducted between October 5, 2020, and April 11, 2021. The survey targeted employed individuals aged 15 and above, with a sample size of approximately 50,000 workers from eligible households across all 17 metropolitan cities and provinces in South Korea. The target population included all employed residents in South Korea at the time of the survey, encompassing workers, business owners, and self-employed individuals. The sampling frame was based on the 2018 Population and Housing Census conducted by the Korean Statistical Information Service.

The sampling method involved stratification by 17 cities/provinces, urban/rural areas, and housing types (apartment/general). Systematic sampling was used, with a two-stage process: first, selecting survey areas proportionally, and second, selecting households within those areas systematically. In multi-person households, one eligible member was randomly chosen using a tablet program.

Face-to-face personal interviews were conducted by professional interviewers using Tablet PC Assisted Personal Interviewing (TAPI). Due to COVID-19 restrictions, there was a pause in fieldwork for about 45 days from December 13, 2020, to January 26, 2021.

For this research, the focus was on regular employees, defined in the survey as those without restrictions on the duration of their employment contract or whose contract was for more than one year, including employees without formal contracts but who underwent standard recruitment processes and were subject to company HR policies or eligible for severance pay.

The choice to focus on regular employees is justified by the need to understand the specific work conditions and perceptions of this significant segment of the workforce. Regular employees, due to their long-term and stable employment status, offer a distinct perspective on workplace dynamics, which is crucial for comprehending broader trends in employee satisfaction and well-being. This approach aligns with recent shifts in employment patterns and the growing importance of understanding the experiences of permanent workers in the evolving labor market.

Table 2 presents demographic and income data of 25,285 subjects from a KWCS. It details gender distribution, showing a near-even split between males (48.9%) and females (51.1%). Age-wise, the largest groups are 30–39 and 40–49 years, accounting for over 50% of the sample. Education levels range from no education to graduate studies, with the majority holding university degrees (39%). Income data categorizes earnings from less than 1 million KRW to over 8 million KRW, with significant portions earning between 2–3 million KRW and 3–4 million KRW. The sector breakdown shows a predominance in the private sector (85.2%).

**Table 2:** Demographics.

Item	Subjects (*N* = 25,285)	Frequency	Percentage
Gender	Male	12359	48.9%
	Female	12926	51.1%
Age	15–19	39	0.2%
	20–29	3127	12.4%
	30–39	6374	25.2%
	40–49	6913	27.3%
	50–59	5959	23.6%
	60–69	2399	9.5%
	70–79	426	1.7%
	80–89	47	0.2%
	90–95	1	0.0%
Education	No education or less than elementary school	60	0.2%
	Elementary school graduation (including special school elementary course)	368	1.5%
	Junior high school graduation (including various schools’ junior high courses)	905	3.6%
	High school graduation (including various schools’ high school courses)	7978	31.6%
	Community college graduation	5156	20.4%
	University graduation	9860	39.0%
	Graduate school or higher	929	3.7%
	Refusal	29	0.1%
Monthly Income (Average net income over the past 3 years, million KRW)	Less than 1 million KRW	632	2.5%
	1 ‐ less than 2	3374	13.3%
	2 ‐ less than 3	7422	29.4%
	3 ‐ less than 4	4528	17.9%
	4 ‐ less than 5	1891	7.5%
	5 ‐ less than 6	821	3.2%
	6 ‐ less than 7	307	1.2%
	7 ‐ less than 8	182	0.7%
	More than 8	171	0.7%
	Unknown/No response	4984	19.7%
	Refusal	973	3.8%
Sector	Private sector	21554	85.2%
	Public sector	2985	11.8%
	Private-Public Cooperative Organization	395	1.6%
	Non-profit organization, Non-governmental organization	302	1.2%
	Others	49	0.2%

### Analysis and results

PLS-SEM was employed in this study due to its suitability for complex model testing, particularly in the context of behavioral studies [99]. PLS-SEM excels in handling predictive models and theory development, especially when the research objective involves both the measurement and structural model [115]. Its ability to efficiently handle smaller sample sizes and complex models with many indicators, as well as its flexibility with data distribution, makes PLS-SEM an ideal choice for analyzing the multifaceted relationships in our study [116].

**Fig 2:**
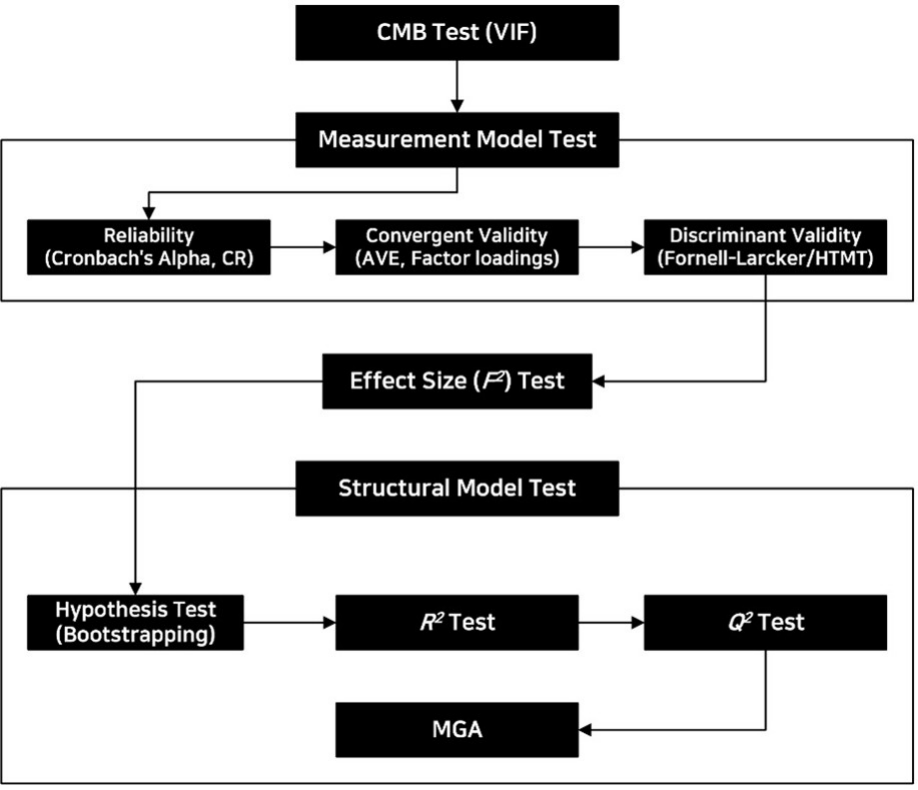
Overall analysis procedure.

The analysis procedure of this study followed a methodical sequence to address the research questions effectively. Initially, the study ensured the quality of the dataset, followed by a preliminary analysis to determine the structure and distribution of the data (Common method bias). The core analysis began with the evaluation of the measurement model to confirm the reliability and validity of the constructs. This involved assessing internal consistency through Cronbach’s Alpha and Composite Reliability, and verifying convergent and discriminant validity through Average Variance Extracted (AVE) and the Fornell-Larcker criterion. These steps established the adequacy of the measurement scales used for the constructs. Following the measurement model validation, the structural model was assessed. The relationships between the constructs were examined using bootstrapping with 5000 resamples to estimate the path coefficients and their significance. This rigorous approach helped to quantify the strength and significance of hypothesized relationships. The analysis concluded with an MGA to explore potential differences in effects across groups defined by different sectoral employment (private vs. public). This provided insights into the contextual variability of the model’s applicability and helped in understanding sector-specific dynamics. The findings from these analytical steps were crucial in drawing conclusions about the factors influencing employee outcomes in varying organizational contexts. Fig 2 illustrates the overall procedure of the analysis.

### Common method bias

In addressing common method bias in this study, we considered the Variance Inflation Factor (VIF) for each construct, as depicted in Table 3. The VIF values, all well below the recommended threshold of 3.3 [117], indicate that multicollinearity is not a concern in our model.

**Table 3:** VIF.

Construct	1	2	3	4	5	6	7	8	9	10
1. Recognition							1.779			
2. Fairness							1.823			
3. Involvement							1.276			
4. Transformational Leadership							1.204	1.004		
5. Overload							1.018	1.017		
6. Competition							1.082	1.015		
7. Engagement										1.005
8. Burnout										1.039
9. Technological Disruption Anxiety										1.032
10. Satisfaction										

### Measurement model

The measurement model of this study is thoroughly evaluated through reliability and convergent validity metrics, as shown in Table 4. Each construct’s reliability is confirmed by Cronbach’s Alpha and Composite Reliability (CR), with values exceeding the recommended threshold of 0.6, indicating strong internal consistency [118]. Convergent validity is established through the Average Variance Extracted (AVE) for each construct, all surpassing the 0.5 benchmark, ensuring that the constructs capture a substantial portion of the variance in the observed variables [119].

**Table 4:** Reliability and convergent validity.

Construct	Item	Mean	St. Dev.	Factor Loading	Cronbach’s Alpha	CR	AVE
Recognition	RCN1	3.406	0.785	0.849	0.716	0.842	0.642
	RCN2	3.452	0.792	0.855			
	RCN3	3.622	0.744	0.688			
Fairness	FAR1	3.538	0.785	0.837	0.713	0.840	0.638
	FAR2	3.607	0.776	0.831			
	FAR3	3.553	0.815	0.722			
Involvement	IVV1	3.182	0.973	0.838	0.762	0.859	0.671
	IVV2	3.024	1.042	0.798			
	IVV3	3.392	0.951	0.821			
Transformational Leadership	TFL1	4.033	0.824	0.919	0.920	0.949	0.861
	TFL2	3.956	0.905	0.925			
	TFL3	3.859	0.920	0.940			
Overload	OVL1	2.780	1.662	0.946	0.866	0.937	0.881
	OVL2	2.789	1.761	0.932			
Competition	COM1	2.805	1.002	1.000	-	-	-
Engagement	EGM1	3.489	0.742	0.885	0.796	0.880	0.710
	EGM2	3.599	0.796	0.878			
	EGM3	3.670	0.804	0.760			
Burnout	BUR1	2.708	0.950	0.958	0.913	0.958	0.920
	BUR2	2.590	0.948	0.960			
Technological Disruption Anxiety	ANX1	2.653	0.957	0.890	0.862	0.912	0.776
	ANX2	2.250	0.827	0.866			
	ANX3	2.231	0.812	0.887			
Job Satisfaction	SAT1	2.927	0.479	1.000	-	-	-
Gender	Gender	0.511	0.500	1.000	-	-	-
Age	AGE	44.380	12.127	1.000	-	-	-

Table 5 presents the Fornell-Larcker criterion results, where the square roots of AVEs (diagonal values) for each construct are greater than their respective inter-construct correlations, confirming discriminant validity. This suggests that each construct is distinct and captures unique aspects of the phenomenon under study [119].

**Table 5:** Fornell-Larcker scale results.

Construct	1	2	3	4	5	6	7	8	9	10
1. Recognition	0.801									
2. Fairness	0.627	0.799								
3. Involvement	0.370	0.408	0.819							
4. Transformational Leadership	0.338	0.365	0.274	0.928						
5. Overload	-0.025	-0.034	-0.001	-0.052	0.939					
6. Competition	0.198	0.073	0.176	0.031	0.115	1.000				
7. Engagement	0.521	0.417	0.286	0.237	0.007	0.155	0.843			
8. Burnout	-0.128	-0.166	0.014	-0.074	0.193	0.148	-0.029	0.959		
9. Technological Disruption Anxiety	-0.057	-0.079	-0.014	-0.074	0.109	0.129	-0.022	0.175	0.881	
10. Satisfaction	0.436	0.316	0.197	0.203	-0.136	0.085	0.316	-0.189	-0.090	1.000

Note: The values on the diagonal represent the square root of AVE.

The Heterotrait-Monotrait Ratio (HTMT) matrix in Table 6 further supports discriminant validity, with values well below the threshold of 0.85, indicating that the constructs are indeed distinct from one another [120].

**Table 6:** HTMT matrix.

Construct	1	2	3	4	5	6	7	8	9	10
1. Recognition										
2. Fairness	0.896									
3. Involvement	0.498	0.544								
4. Transformational Leadership	0.424	0.451	0.323							
5. Overload	0.035	0.043	0.035	0.058						
6. Competition	0.229	0.086	0.207	0.033	0.124					
7. Engagement	0.683	0.548	0.350	0.275	0.026	0.169				
8. Burnout	0.158	0.206	0.047	0.080	0.216	0.155	0.055			
9. Technological Disruption Anxiety	0.067	0.101	0.045	0.082	0.130	0.146	0.025	0.203		
10. Satisfaction	0.511	0.375	0.222	0.212	0.145	0.085	0.347	0.198	0.091	

Lastly, Table 7 shows the effect sizes (*f*^*2*^), which provide insights into the relative impact of the independent variables on the dependent variables. These values indicate the magnitude of the constructs’ influence in the model, contributing to a comprehensive understanding of the relationships among the constructs [121].

**Table 7:** Effect size (*f*^*2*^).

Construct	1	2	3	4	5	6	7	8	9	10
1. Recognition										
2. Fairness	0.896									
3. Involvement	0.498	0.544								
4. Transformational Leadership	0.424	0.451	0.323							
5. Overload	0.035	0.043	0.035	0.058						
6. Competition	0.229	0.086	0.207	0.033	0.124					
7. Engagement	0.683	0.548	0.350	0.275	0.026	0.169				
8. Burnout	0.158	0.206	0.047	0.080	0.216	0.155	0.055			
9. Technological Disruption Anxiety	0.067	0.101	0.045	0.082	0.130	0.146	0.025	0.203		
10. Satisfaction	0.511	0.375	0.222	0.212	0.145	0.085	0.347	0.198	0.091	

### Hypothesis test

The structural model of this study was rigorously assessed to understand the relationships between the constructs. The analysis, based on 5,000 resampling bootstraps in PLS-SEM, provides the path coefficients, *t-*values, and *p-*values for each hypothesis, as detailed in Table 8.

**Table 8:** Summary of the results.

H	Predictor	Outcome	*β*	*T*	*P*	Hypothesis
H1	Recognition	Engagement	0.394	52.621	0.000	Supported
H2	Fairness	Engagement	0.123	16.286	0.000	Supported
H3	Involvement	Engagement	0.070	11.062	0.000	Supported
H4a	Transformational Leadership	Engagement	0.039	5.991	0.000	Supported
H4b	Transformational Leadership	Burnout	-0.069	10.829	0.000	Supported
H5a	Overload	Engagement	0.017	3.000	0.001	Not Supported (Significant)
H5b	Overload	Burnout	0.174	26.985	0.000	Supported
H6a	Competition	Engagement	0.052	9.076	0.000	Supported
H6b	Competition	Burnout	0.130	19.674	0.000	Supported
H7	Engagement	Satisfaction	0.307	48.022	0.000	Supported
H8	Burnout	Satisfaction	-0.176	28.279	0.000	Supported
H9	Technological Disruption Anxiety	Satisfaction	-0.054	9.167	0.000	Supported
CV	Gender	Satisfaction	0.097	8.267	0.000	Significant
CV	Age	Satisfaction	-0.055	9.247	0.000	Significant

Note: CV stands for control variable.

The model’s predictive power, indicated by the *Q*^*2*^ values in Table 9, confirms the model’s practical relevance. Engagement shows a strong predictive power (*Q²* = 0.294), suggesting the model’s effectiveness in explaining variations in employee engagement. Burnout and job satisfaction also demonstrate acceptable predictive capabilities with *Q*^*2*^ values of 0.058 and 0.132, respectively.

**Table 9:** Predictive power (*Q*^*2*^).

Construct	Q^2^predict	RMSE	MAE
Engagement	0.294	0.841	0.652
Burnout	0.058	0.971	0.797
Job Satisfaction	0.132	0.932	0.526

### Multi-group analysis

The MGA of this study, comparing the private and public sectors, reveals significant differences in the effects of various workplace factors on employee outcomes. Notably, Transformational Leadership shows a more significant reduction in burnout in the public sector than in the private sector, as indicated by a negative difference (-0.064) with a significant *t-*value (3.260) and *p-*value (0.001). This suggests that transformational leadership practices are more effective in mitigating burnout in the public sector. Overload’s impact on Burnout also shows a significant difference between sectors, with a higher negative impact in the public sector, indicating that workload pressures contribute more to burnout in this environment. In contrast, Competition’s effect on Burnout is significantly more pronounced in the private sector *(p-*value < 0.001), highlighting the higher stress and burnout associated with competitive environments in these workplaces. Furthermore, the impact of burnout on job satisfaction shows a more negative effect in the public sector, emphasizing the stronger influence of burnout on reducing job satisfaction there. Additionally, age’s effect on Satisfaction reveals a significant difference, with a more negative impact in the private sector, indicating that age-related factors more substantially affect job satisfaction in this sector. Table 10 show the results of MGA.

**Table 10:** The MGA results (private sector–Public sector).

H	Cause	Effect	*β*_*private*_	*β*_*public*_	Difference	*T*	*P*	Sig.
H1	Recognition	Engagement	0.391***	0.415***	-0.024	1.017	0.155	NS
H2	Fairness	Engagement	0.123***	0.113***	0.010	0.426	0.335	NS
H3	Involvement	Engagement	0.071***	0.066***	0.005	0.237	0.406	NS
H4a	Transformational Leadership	Engagement	0.041***	0.021	0.021	1.032	0.151	NS
H4b	Transformational Leadership	Burnout	-0.059***	-0.123***	0.064	3.260	0.001	Sig.
H5a	Overload	Engagement	0.016**	0.026	-0.010	0.569	0.285	NS
H5b	Overload	Burnout	0.167***	0.217	-0.050	2.523	0.006	Sig.
H6a	Competition	Engagement	0.052***	0.058	-0.007	0.374	0.354	NS
H6b	Competition	Burnout	0.141***	0.071	0.069	3.360	0.000	Sig.
H7	Engagement	Satisfaction	0.299***	0.317	-0.018	0.893	0.186	NS
H8	Burnout	Satisfaction	-0.166***	-0.237	0.071	3.616	0.000	Sig.
H9	Technological Disruption Anxiety	Satisfaction	-0.048***	-0.053**	0.005	0.294	0.384	NS
CV	Gender	Satisfaction	0.100***	0.044	0.056	1.536	0.062	NS
CV	Age	Satisfaction	-0.065***	-0.028*	-0.036	1.989	0.023	Sig.

Note: CV stands for ‘control variable.’ NS stands for ‘not significant.’

*: p < .05

**: p < .01

***: p < .001.

## Discussion

### Main path

The discussion of this study’s findings offers a critical examination of how various workplace factors influence employee engagement, burnout, and overall job satisfaction. By comparing our results with existing literature, we aim to provide a comprehensive understanding of these dynamics and their implications in the modern workplace.

The findings show that the substantial positive effect of recognition on engagement (*β =* 0.394), strongly aligning with Herzberg [18]’s Two-Factor Theory, emphasizing recognition as a fundamental motivator enhancing employee satisfaction and productivity. This result is reinforced by research from Kooij, Tims [22], who found that recognition significantly boosts employee engagement, underscoring its importance as a key driver in the workplace. The strong influence of recognition on engagement among regular employees emphasizes the psychological impact that feeling valued and acknowledged has in the workplace. When employees perceive their efforts and achievements are recognized, it fosters a sense of worth and validation, which in turn fuels their motivation and commitment to their tasks. This process not only enhances their immediate job involvement but also contributes to a broader sense of connection and loyalty to the organization, reinforcing the idea that recognition is a powerful tool in shaping positive employee attitudes and behaviors.

Fairness’s impact on engagement (*β =* 0.123) supports the concept of organizational justice as proposed by Greenberg [6]. Although significant, its comparatively weaker influence than recognition highlights fairness as an essential, yet not the sole, determinant of employee engagement. This finding portrays fairness as a foundational factor that positively influences work outcomes, including engagement, but is not the exclusive contributor. When regular employees perceive that policies, resource distribution, and interpersonal interactions are fair, it enhances their trust and commitment to the organization, leading to higher levels of active participation and enthusiasm in their roles.

Involvement’s positive but modest effect on engagement (*β =* 0.070) corroborates the significance of employee involvement in decision-making processes, aligning with former studies [122, 123]. This finding resonates with the empowerment theory, suggesting that while involvement positively impacts engagement, it is not the sole influential factor, as indicated by its lower coefficient compared to recognition. This suggests that involvement is a beneficial, yet not dominant, contributor to employee engagement.

Transformational leadership’s dual impact on engagement and burnout, with a positive influence on engagement (*β =* 0.039) and a negative impact on burnout (*β =* -0.069), aligns with the extant literature [8, 54]. Transformational leadership’s dual impact reflects its multifaceted role in shaping employee experiences in the workplace. Its positive influence on engagement, albeit moderate, suggests that inspirational, supportive, and motivating leadership behaviors can enhance employees’ enthusiasm and commitment to their work [53]. On the flip side, the negative impact on burnout highlights the protective role transformational leaders play in mitigating workplace stressors, thereby preserving employee well-being. This dual effect underscores the significance of transformational leadership in not only fostering a more engaged workforce but also in reducing the detrimental effects of burnout.

The relationship between overload and engagement presents a nuanced perspective in organizational studies. Contrary to the traditional view that overload invariably reduces engagement by sapping employee energy and motivation, this study’s findings suggest a slight positive correlation (*β =* 0.017), albeit not strong enough to support the hypothesis. This suggests that, under certain conditions, increased demands might stimulate a heightened level of engagement through mechanisms akin to Csikszentmihalyi [124]’s Flow theory, where challenges become enjoyable and enriching. However, these results deviate from traditional interpretations as seen in previous studies, which link higher demands directly with reduced job satisfaction and increased stress [68–70]. This discrepancy invites further investigation into the conditions under which workload might act as a catalyst for engagement rather than a deterrent, potentially exploring the roles of individual resilience and workplace support in transforming potential stressors into sources of engagement. On the other hand, the stronger association of overload with burnout (*β* = 0.174) reflects the JD-R’s assertion [14] that while short-term challenges can boost engagement, prolonged overload is likely to lead to burnout. The positive relationship between workload overload and employee engagement suggests that up to a certain point, increased job demands can stimulate employees, potentially leading to a heightened sense of achievement and fulfillment in their work. However, this relationship is nuanced, indicating that while moderate overload can be engaging, excessive demands could lead to negative outcomes like stress or burnout.

Competition’s dual impact on engagement and burnout, with a positive effect on engagement (*β =* 0.052) and a significant increase in burnout (*β =* 0.130), highlights its complex nature in the workplace. This dual-edged effect indicates that while competition can drive engagement through goal-setting and achievement, it also significantly elevates stress and burnout, as identified in former studies [78, 125].

The strong positive effect of engagement on satisfaction (*β =* 0.307) reflects the findings of Harter et al. [82], indicating that high levels of engagement are closely linked to greater job satisfaction. Conversely, the negative effect of burnout on satisfaction (*β =* -0.176) is in line with Maslach and Leiter [70]’s research, suggesting that burnout significantly undermines job satisfaction, eroding the positive aspects of work life.

The significant negative relationship between burnout and job satisfaction (*β* = -0.176) strongly corroborates the extensive literature on the deleterious effects of burnout on an employee’s sense of job fulfillment and well-being [126–128]. As supported by previous studies [91, 93, 129], prolonged exposure to job stressors, leading to burnout, can drastically reduce job satisfaction by depleting personal and psychological resources. This relationship indicates that as burnout intensifies, characterized by emotional exhaustion, depersonalization, and a reduced sense of personal accomplishment, it profoundly diminishes satisfaction, reflecting the critical need for effective workplace strategies to manage and mitigate burnout to maintain high levels of employee satisfaction.

The negative impact of technological disruption anxiety on satisfaction (*β =* -0.054) highlights the stress and uncertainty caused by technological changes in the workplace. This finding suggests that anxiety about technological disruption and its implications can significantly diminish overall job satisfaction. This anxiety, stemming from concerns about job security, the need for new skills, or changes in job roles, can significantly diminish an employee’s overall satisfaction with their job. It highlights the importance of managing and addressing these anxieties proactively to maintain a stable and satisfied workforce in an era of continuous technological advancement.

The significant influence of gender (*β =* 0.097) on satisfaction might reflect unique workplace experiences based on gender, suggesting differing perceptions and reactions to workplace dynamics. The negative impact of age on satisfaction (*β =* -0.055) indicates varying satisfaction levels across age groups, which might be attributed to diverse job expectations, career stages, or generational differences in work values and attitudes.

### Difference between private sector and public sector

The MGA conducted in this study, focusing on Korean regular employees in the private and public sectors, reveals some significant insights. While several relationships did not show significant differences between these sectors, notable findings emerged in other areas.

Firstly, the effect of transformational leadership (H4b) on burnout shows a significant difference between sectors, with a more negative coefficient in the public sector (-0.123) compared to the private sector (-0.059). This suggests that transformational leadership is more effective in mitigating burnout in the public sector. This could be due to the structured nature of public sector organizations, where transformational leaders can significantly influence employee motivation and well-being by offering inspiration, support, and a clear vision. The effectiveness of transformational leadership in the public sector highlights the importance of leadership development programs that focus on transformative qualities to effectively combat employee burnout and enhance overall job satisfaction.

Similarly, overload’s (H5b) impact on burnout is significantly different, with the public sector showing a higher coefficient (0.217) than the private sector (0.167). This indicates that workload overload contributes more to burnout in the public sector, underlining the importance of workload management, especially in public sector organizations. This could reflect the unique challenges and stressors present in public sector jobs, such as bureaucratic constraints and high job demands with potentially fewer resources for coping. The finding emphasizes the need for targeted strategies in the public sector to manage workload effectively, provide adequate support, and create a sustainable work environment to mitigate the heightened risk of burnout due to overload.

The significant result for hypothesis H6b indicates that competition has a more substantial effect on burnout in the private sector than in the public sector, as evidenced by a higher coefficient in the private sector (0.141) compared to the public sector (0.071). This suggests that competitive pressures and dynamics in the private sector are more likely to contribute to employee burnout, possibly due to a more intense focus on performance and results in these environments. The notable difference in coefficients and its statistical significance highlight the need for private sector organizations to carefully manage competitive elements within the workplace to mitigate the risk of burnout among employees.

The impact of burnout on job satisfaction (H8) also varies significantly between sectors, with a much more negative effect in the public sector (-0.237) than in the private sector (-0.166). This suggests that burnout substantially decreases job satisfaction among public sector employees more than in the private sector. This heightened impact could be due to the unique stressors and challenges in the public sector, such as less autonomy, higher job demands, and possibly lower levels of job resources, which might exacerbate the adverse effects of burnout on job satisfaction. This significant difference highlights the critical need for effective burnout management strategies in the public sector, focusing on enhancing job resources, employee support, and work-life balance to improve overall job satisfaction.

Finally, the effect of age on satisfaction shows a significant difference, with a more negative coefficient in the private sector (-0.065) than in the public sector (-0.028), indicating that age-related factors have a more substantial impact on job satisfaction in the private sector. This could imply that age-related factors, such as career progression, work-life balance, or evolving job expectations, might impact satisfaction levels more significantly in the private sector. The finding underscores the need for private sector organizations to address the specific needs and expectations of their aging workforce, ensuring strategies are in place to maintain or enhance job satisfaction across different age groups.

## Conclusion

This study extends the current understanding of organizational behavior by exploring the intricate dynamics between various workplace factors and employee outcomes. Unlike previous studies that have often treated factors like recognition, fairness, and leadership style in isolation, our research uniquely investigates their combined impact on employee engagement, burnout, and satisfaction. For instance, while the motivational influence of recognition has been well-documented in the literature [18, 22], our study contributes by quantifying its substantial impact and comparing it with other factors like fairness and involvement. This comparison offers a more comprehensive understanding of their relative significance in influencing employee engagement. Moreover, our findings on the dual impact of transformational leadership extend beyond the typical focus on positive outcomes [3], by also highlighting its role in mitigating burnout. This dual perspective fills a gap in existing literature, which has not fully explored the negative relationship between transformational leadership and burnout. The nuanced findings regarding the positive association between workload overload and engagement offer a fresh perspective on the complex relationship between job demands and employee motivation, challenging the conventional notion that overload is uniformly detrimental. For scholars, these findings suggest the need for more integrated models of organizational behavior that consider multiple factors simultaneously. Future research could explore the interplay between these factors in different organizational contexts or industries, offering a more tailored understanding of their impacts.

The findings of this study offer actionable insights for practitioners across various organizational levels, from top managers and industry leaders to middle managers and employees. Understanding the dynamics of workplace factors and their impact on employee engagement, burnout, and satisfaction is crucial for fostering a healthy and productive work environment. For top managers and industry leaders, the significant role of recognition in enhancing employee engagement cannot be overstated. Companies should prioritize implementing recognition programs that acknowledge employees’ efforts and achievements. For example, a tech company might establish an ‘Innovator of the Month’ award to highlight and reward creative contributions [130]. This not only boosts morale but also encourages a culture of innovation and excellence. Middle managers can play a pivotal role in ensuring fairness and involvement within their teams. They should aim for transparent communication and equitable resource distribution to enhance team members’ sense of fairness. For instance, in a sales team, ensuring that leads are distributed evenly and performance metrics are transparent can contribute significantly to a sense of fairness, thereby increasing engagement [131]. Industry leaders are positioned to address the negative impact of technological disruption anxiety on job satisfaction. In industries undergoing rapid technological change, such as the automotive or IT sectors, leaders should invest in continuous learning and development programs. By providing employees with training and upskilling opportunities [132], organizations can mitigate anxiety and improve adaptation to technological changes, thus preserving job satisfaction. At the employee level, understanding the dual impact of workload and competition on both engagement and burnout is vital. Employees should be encouraged to seek a balance, taking on challenges to boost engagement while being mindful of the potential for burnout. Organizations could implement wellness programs or provide resources for stress management to help employees navigate these challenges effectively.

This study, while comprehensive, has limitations that open avenues for future research. One such limitation is the cross-sectional nature of the study, which restricts our ability to make causal inferences. Future research could employ longitudinal or experimental designs to better understand the causal relationships between these workplace factors and employee outcomes. Additionally, this study focused on regular employees; thus, exploring these dynamics among different employee categories, such as temporary or part-time workers, could offer a broader perspective. Future research might also consider the impact of cultural differences on these relationships, as cultural contexts could significantly influence how employees perceive recognition, fairness, and leadership.
